# Soft tissue foreign bodies: A training manual for sonographic diagnosis and guided removal

**DOI:** 10.1002/jcu.22856

**Published:** 2020-05-08

**Authors:** Veronica J. Rooks, William E. Shiels, James W. Murakami

**Affiliations:** ^1^ Department of Radiology Tripler Army Medical Center Honolulu Hawaii USA; ^2^ Deceased, Prior Active Duty Army, Department of Radiology Nationwide Children's Hospital Columbus Ohio USA; ^3^ Department of Radiology Nationwide Children's Hospital Columbus Ohio USA

**Keywords:** foreign bodies, localization, removal, soft tissues, ultrasonography

## Abstract

Sonography provides excellent detection, localization, and characterization of soft‐tissue foreign bodies. Ultrasound guided foreign body removal is a safe and highly successful minimally invasive procedure that facilitates effective treatment and avoidance of complications in patients with soft tissue foreign bodies. Focused laboratory training is critical to successful implementation of a sonographic foreign body management practice.

## 1 INTRODUCTION

Ballistic foreign bodies (FB) in current combat scenarios, to include injuries sustained with improvised explosive devices (IED), consist of materials such as metal, ceramic, stone, wood, plastic, clothing, flesh, bone, or vegetable matter. Ballistic FB such as wood, plastic, clothing, flesh, or vegetable matter that are not visible with radiographic or fluoroscopic evaluation may be identified and removed with ultrasound guidance.^1^, ^2^, ^3^, ^4^, ^5^, ^6^

As a general rule, any soft‐tissue FB requires removal when they become symptomatic or develop infectious complications or as no longer desired. Many superficial FB can be palpated and readily removed. When superficial abscesses develop an incision over the area often results in expulsion of both the contained pus and the FB.

Surgeons, emergency physicians, and primary care physicians acknowledge the futility of blind removal attempts of small and deep foreign bodies. Similarly, physicians are familiar with the limitations of fluoroscopic guidance for removal of radiopaque foreign bodies: radiation exposure and the difficulty imposed by two‐dimensional (2D) visualization on the fluoroscope.

High resolution sonography is an excellent imaging tool used for detection, localization and removal of nonradiopaque soft‐tissue FB.^1^, ^2^, ^3^, ^4^ Radiopaque FB are frequently detected and grossly localized with plain radiographs. Sonography plays an expanding role in identification, characterization, and detailed three‐dimensional (3D) localization with respect to vital neurovascular structures and tendons.

This training guide will focus on management of soft‐tissue FB, including those in muscle, tendon, and intra‐articular spaces and bony structures.

## 2 SONOGRAPHIC DETECTION, LOCALIZATION, AND CHARACTERIZATION OF FOREIGN BODIES

Sonography is most frequently used for primary detection, localization, and characterization of nonradiopaque FB, following initial evaluation with plain radiography. Occasionally, computed tomography or magnetic resonance imaging are performed in the evaluation of an unresponsive soft‐tissue infection, with sonography providing more accurate detection, real time 3D localization, and characterization of the offending FB.

Detection of soft‐tissue FB is performed with the highest resolution sonographic transducers available. In the search for embedded FB, high frequency linear array transducers (7‐18 MHz) are the most useful in detection of radiopaque and nonradiopaque FB, ranging in size from 0.5 to more than 10 mm in transverse dimension. Meticulous scanning is critical for detection, since minimally echogenic FB can be sonographically subtle and mistaken as a muscle fibril or fascial tissue plane. Other distractions may include gas, proteinaceous of echogenic fluid, or multiple small foreign bodies.

In the initial search process, FB are most easily detected when the sound beam strikes the object aligned with the FB longitudinal axis, delineating the full length of the FB. Frequently, initial sonography will strike in the transverse plane to the plane of the FB, defining small linear FB as more echogenic than surrounding soft tissues.

**FIGURE 1:**
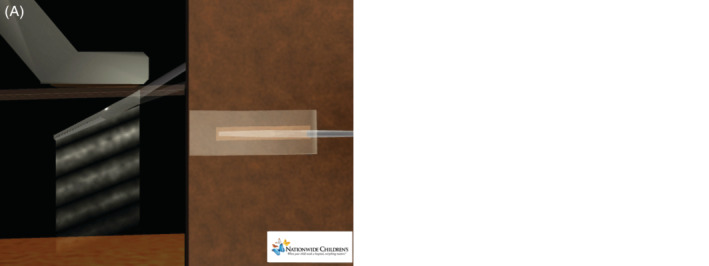
Oblique cross‐cut artifact during forceps management. A, Correct longitudinal alignment of forceps in the long axis of the sound beam. B, Transducer pivot foreshortens visualized portions of the forceps, losing visualization of the jaw forceps tips and proximal shaft of the forceps causing cross‐cut artifact

Once an FB is detected, it is important to maintain alignment of the sound beam with the long axis of the FB for conspicuity and definition. Caution is advised to the potential pitfall of the “oblique cross‐cut” artifact created by scanning obliquely to the true long axis of the FB, foreshortening the appearance of the FB. The same concept of pitfall of oblique cross‐cut artifact is applicable to accurate visualization of the forceps during FB removal (see Figure 1, which shows oblique cross‐cut artifact during forceps management; see Video S1, demonstrating oblique cross‐cut artifact). Once fully defined, the operator can accurately localize the FB with respect to surrounding structures.

The response of surrounding tissues has diagnostic significance, as granulation tissue and pus purulent debris will create hypoechoic zones.^1^, ^2^, ^3^, ^4^ Longstanding inflammation surrounding a FB will produce a hypoechoic “halo” of granulation tissue, with vascular in‐growth demonstrated with color Doppler sonography. If a surgeon or other physician has unsuccessfully attempted removal, there is often air in the surrounding soft tissues, limiting definition of the retained object immediately following surgical dissection. It is prudent to dress the wound and allow a few days to pass for air absorption, prior to repeat sonographic examination.

## 3 LOCALIZATION PRIOR TO OPEN SURGICAL REMOVAL

Localization may include preoperative verbal definition of anatomic coordinates for a surgeon and/or preoperative skin marking for surgical mapping. An additional means of preoperative localization is placement of localization wires with sonographic guidance. Localization needles are placed so that ideally two hooked wires are deployed immediately deep to the FB, one deep to the proximal tip and one deep to the distal tip of the FB. The surgeon can follow the wire and encounter the FB prior to reaching the hook. Localization wire placement is performed following generous administration of local anesthesia under sonographic guidance, to the depth of wire hook placement. Once the localization wire is in place, the placement needle is removed and the wires are secured to the skin with a sterile dressing. The hook type wire provides a small target for surgical dissection and is easily fixed in surrounding soft tissues under ultrasound guidance. After infiltrating the operative bed, local anesthetic can be injected adjacent to the FB, clearly defining the FB from surrounding soft‐tissues. The patient can then be transferred to the surgical suite for open resection.

## 4 FOREIGN BODY REMOVAL

### 4.1 Operator ergonomics and patient set up

Prior to FB removal, consideration of operator ergonomics and proper patient positioning is crucial to maximal ultrasound guided foreign body removal (USFBR) success. Dim lighting improves visualization of the ultrasound screen. Patient location is best when the ultrasound screen is in front of the operator and the patient is between the operator and the ultrasound unit. The location of the FB within the patient may require creative patient positioning, so that the transducer is positioned upright, perpendicular to the floor of the room, and parallel to the operator. Having the ultrasound screen approximately 3 to 4 ft from the operator is comfortable for visualization and adjustment of ultrasound machine technical parameters. Many operators prefer to perform USFBR while sitting, reducing ergonomic fatigue. The table height should be adjusted to avoid the operator leaning over the patient, thus preventing low back strain.

Local anesthesia is critical during FB removal procedures. With proper technique, 25 to 30 gauge needles are visualized for deposition of deep local anesthesia to the level of the FB. First, deep local anesthesia is administered with sonographic guidance in the operative pathway of hemostat dissection and FB removal. Immediately prior to 4 to 5 mm skin incision with a #11 scalpel blade and forceps dissection, a second bolus of anesthetic is administered at the tip and along the body of the FB. This anesthetic bolus serves to hydrodissect the surrounding soft tissues away from the FB, whether it be a toothpick, gravel, or brass BB, facilitating tactile sensation forceps contact, grasp, and removal.^2^, ^4^, ^7^, ^8^ (see Videos S2 and S3, demonstrating hydrodissection).

### 4.2 Technical considerations

Interrogation of adjacent soft‐tissues is essential for the pre‐procedural decision‐making and planning process. Foreign bodies located adjacent and deep to nervous or vascular structures may not be accessible for USFBR and may require open surgical removal. In general, foreign bodies need not be removed unless symptomatic. Wood fragments and or glass shards should not be attempted to be removed unless there is pain or infection at the site. Foreign bodies removed within tendons or tendon sheaths require coordination and close follow‐up. In situations where a retained FB has associated infectious tenosynovitis, close coordination with the respective surgeon is mandatory, with some surgeons preferring open FB removal and wound debridement. Fine motor control during USFBR may be best accomplished with the transducer manipulated by the operator's nondominant hand and the forceps controlled by the dominant hand. The optimal hand grasp and transducer movement is described as “contact scanning.”^2^ In contact scanning, the transducer is grasped at the base with four fingers, with the fifth finger lying flat on the patient's skin, maintaining “contact” with the patient during transducer movements. Contact scanning serves multiple purposes: reduces forearm and hand fatigue; maintains proper transducer pressure and eliminates pain caused by excessive transducer force; improves hand‐eye‐transducer coordination; and facilitates fine motor control and stability during USFBR procedures.

**FIGURE 2:**
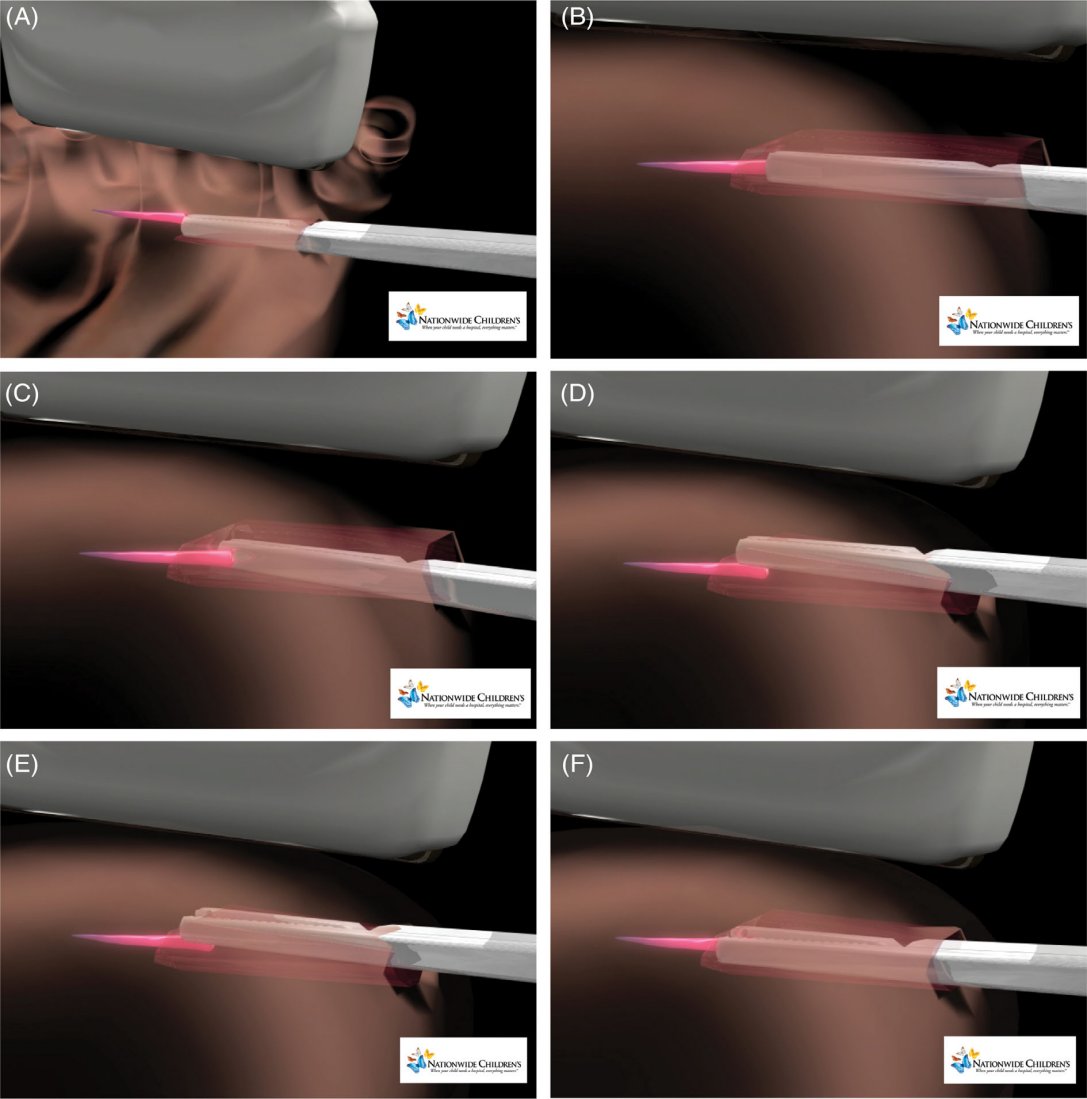
Horizontal forceps alignment removal steps. A, Closed forceps approaching with blunt dissection and contacting the foreign body tip. B, Closed forceps defining the left margin of the foreign body. C, Closed forceps defining the right margin of the foreign body. D, Closed forceps returning to the top of the foreign body. E, Forceps open over the center point of the foreign body. F, Forceps lowered in position for grasp and removal of foreign body

**FIGURE 3:**
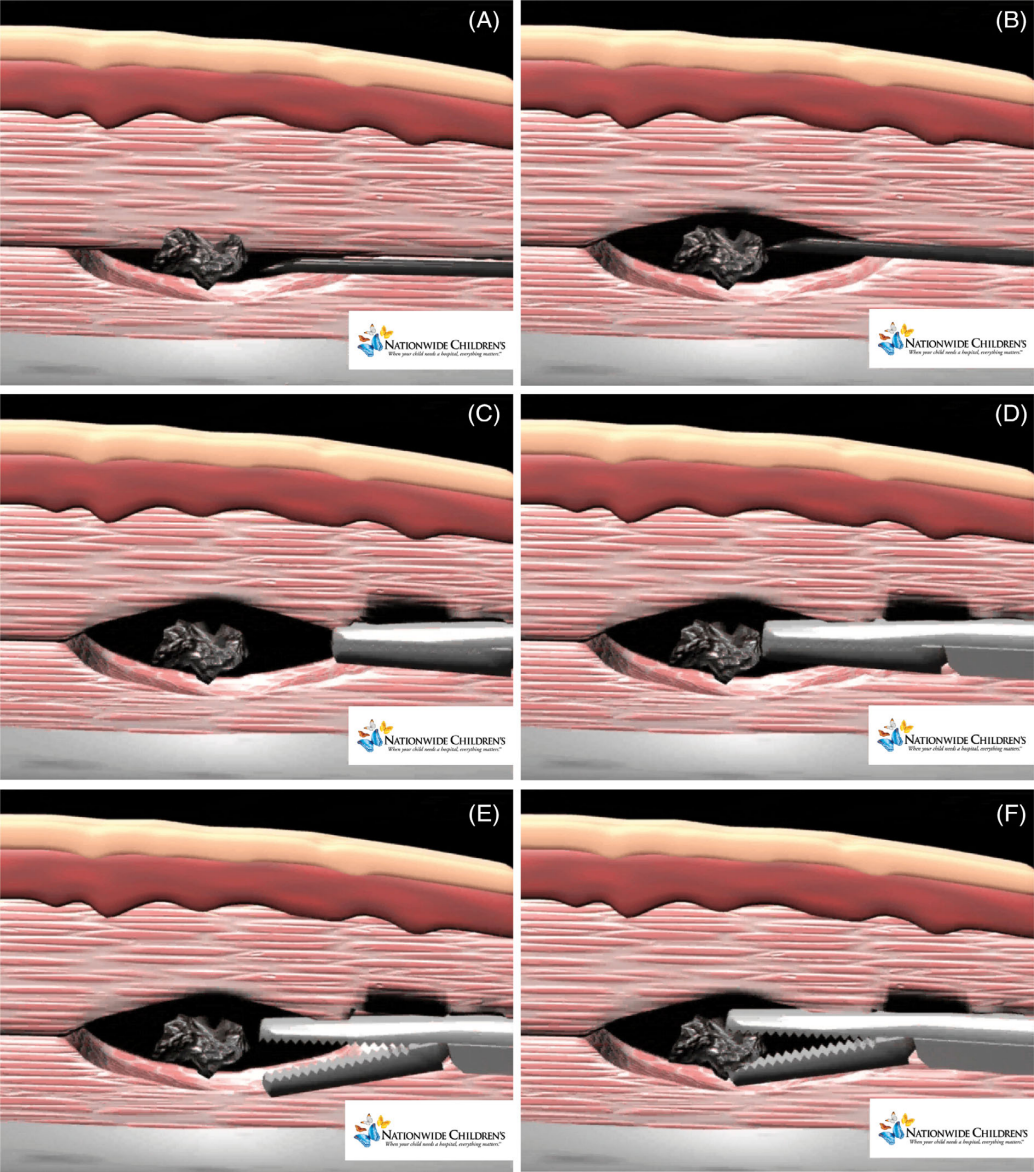
Vertical forceps alignment removal steps. A, Needle in position for lidocaine hydrodissection injection below the tip of the foreign. B, Needle in position for lidocaine hydrodissection injection above the tip of the foreign body. C, Closed forceps with blunt dissection approaching the foreign body. D, Closed forceps contacting the tip of the foreign body. E, Forceps opened to move forward and grasp the tip of the foreign body. F, Forceps jaws firmly closed on the foreign body for removal

USFBR is performed with freehand sonography. Needle guides are not appropriate in USFBR due to the required instrumentation and the need for lateral movements. The most successful forceps approaches are perpendicular or shallow oblique to the sound beam. The forceps may be introduced horizontal or vertical (see Figures 2 and 3, which show horizontal and vertical forceps alignment removal steps). USFBR is performed with the highest possible transducers (7‐18 MHz); linear array transducers provide a flat field and sonographic match with straight forceps. In small surface area operating fields such as the heel, hand, toes, neck, face, and/or orbit, small footprint compact linear array (hockey‐stick) transducers are ideal, facilitating fine motor control.

**FIGURE 4:**
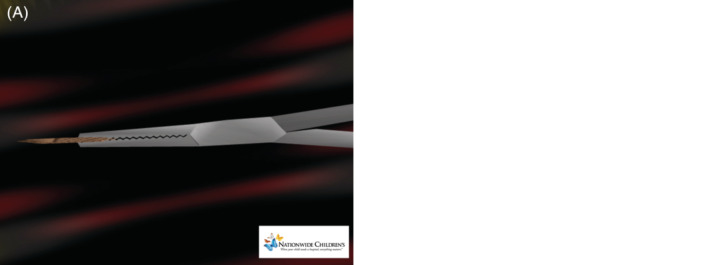
Volume averaging artifact. A, Foreign body aligned with forceps with appearance of good alignment but no tactile sensation of grasp or successful removal. B. Oblique view of the incorrect alignment of the forceps and foreign body illustrating adjacent position of the forceps and foreign body allowing sonographic volume averaging artifact

Forceps selection is also important for optimal technical success. The standard forceps provide the best forceps working surface area and design for USFBR; the straight forceps are preferred over curved forceps. Thin, tapered forceps are useful in deep fields of operation, since the articulation of the forceps occurs deep in a wound and may be manipulated through small incisions (4‐5 mm) large enough for the body of the slender forceps to pass. Thin tapered forceps have also proven useful in removal of small and fragile FB, reducing the chance for fragmentation. Commercially assembled kits include both forceps with serrated jaws and needle drivers with smooth jaws. When removing smooth metal FB, serrated forceps are preferred due to the friction of the jaws during grasp. USFBR is performed with sterile technique, including sterile transducer covers. Once the path of removal is selected, the FB is aligned in the long axis of the sound beam and positioned eccentrically, off to the side of the sonographic field, opposite the forceps. The eccentric position provides up to 50% of the sonographic field for visualization of the forceps during the blunt dissection approach and removal steps (see Video S4, demonstrating this approach on a turkey breast model). The tips of the forceps must remain closed during blunt dissection until contact with the FB, avoiding unnecessary grasp and crushing of soft tissues in the dissection field. If hydrodissection and/or blunt dissection define granulation or fibrous tissue encasing a FB, sharp dissection is required to cut the encasing tissue from the tip of the FB to allow forceps dissection, contact, and successful removal. Options for sharp dissection include #11 scalpel blade or a large bore needle (12G), performing sharp dissection of the granulation or fibrous tissue under real‐time sonographic guidance (see Figure 4, which shows volume averaging artifact).

Prior to opening the forceps jaws, the edge of the FB is contacted and a tap or tactile sensation is felt in the operator's fingertips (see Video S5, demonstrating proper tactile contact with splinter prior to opening forceps). Metallic, stone, and glass have a crisp sensation, sometimes even audible as the forceps grasp. Wood and plastic have a more subtle, soft feel when contacted and grasped. Once jaws are felt closing on the FB, firm jaws closure is maintained during removal. Additionally, detecting resistance from surrounding tissues during attempted removal processes is critical to avoiding unnecessary tissue damage.

USFBR is most quickly accomplished with the sound beam long axis aligned with the long axis of the forceps and FB.

If difficulty is encountered in the longitudinal plane, the transducer may be rotated into a transverse plane. In the transverse sectional plane, the forceps tips can be observed and adjusted accordingly to grasp to FB. Once grasped, real‐time sonography monitors foreign body removal to avoid fragmentation. Following removal, the remaining soft tissue field is sonographically interrogated to evaluate for retention of small fragments. Wound care after removal follows traditional wound closure and management. USFBR is most often performed with 4 to 5 mm incisions, thus sutures are not required. If USFBR is performed in the setting of an established wound infection or abscess, the wound is irrigated with copious amounts of saline lavage, dressed with a sterile dressing, and is allowed to heal by secondary intention. Oral antibiotics are routinely administered to treat minor puncture wound infections established prior to USFBR, if the FB removed is not a medical device.

### 4.3 Pitfalls

USFBR is technically challenging thus hands on simulation training is extremely valuable prior to attempting this procedure on patients. Tissue simulators provide simulation of tissue resistance, steps required for successful USFBR, as well as prospective demonstration of pitfalls that can be anticipated and managed in clinical situations. Preparation of the training tissue simulators includes embedding the foreign bodies with the simulator submerged under water, avoiding introduction of air during preparation.

Intraoperative pitfalls include volume averaging artifacts, suboptimal forceps positioning, grasp position, and overly aggressive grasping techniques. With volume averaging, forceps jaws that are adjacent to one side of the FB can be summated to appear in good position for removal; however, upon opening the tips, the operator is not grasping the foreign body (see Figure 4, which shows volume averaging artifact) (see Videos S5 and S6 demonstrating proper and improper alignment). This pitfall is best managed with careful tactile definition of the top and sides of the foreign body prior to returning to the top of the FB for attempted removal. The fourth dimension is the contact of the foreign body with the forceps as felt by the operator. When the forceps comes into contact with the foreign body, there is a tactile sensation that is transmitted back through the forceps to the operator and the operator will feel that the foreign body is present at the tip of the forceps. Operators should train to be proficient with both horizontal and vertical forceps jaws orientation, as both skill sets are useful during USFBR.

The second pitfall is blunt dissection and approach of the FB with open forceps jaws prior to foreign body contact. This pitfall results in unnecessary tissue injury and impedes both foreign body grasp and removal. The third pitfall is referred to as “central grasp.” Central grasp occurs when the foreign body is grasped in the middle and results in the fracture of FB, converting a single foreign body removal into a multiple FB procedure. Similarly, the fourth forceps pitfall, “aggressive grasp,” can crush a fragile FB and multiply the number of foreign bodies for removal. During the grasp and removal stages of USFBR, gentle and meticulously controlled finger pressure must be maintained to avoid excessive grasp force.

The last forceps pitfall occurs when FB near joints are encountered. Blunt dissection is pursued but the tip of the foreign body cannot be contacted or grasped due to intra‐articular positioning and the joint capsule is found interposed between the FB and the forceps. Intra‐articular foreign body removal can be successfully completed, entering the joint with ultrasound guided arthrotomy using a #11 scalpel blade under direct sonographic guidance.

## 5 CONCLUSION

Ballistic and nonballistic injuries with embedded soft‐tissue FB are challenging clinical dilemmas. This technique can also be used to remove implanted medical devices. Sonography provides excellent detection, localization, and characterization of soft‐tissue foreign bodies. USFBR is a safe and highly successful minimally invasive procedure that facilitates effective treatment and avoidance of complications in patients with soft tissue foreign bodies. Focused hands on simulation training is critical to successful implementation of a sonographic foreign body management practice.

## Supporting information


**Video S1**. Oblique cross‐cut artifact using sonography.

**Video S2**. Hydrodissection surrounding gravel with forceps removal.

**Video S3**. Hydrodissection around toothpick with tactile sensation of forceps prior to removal.

**Video S4**. Blunt dissection approach on a turkey breast model.

**Video S5**. Proper alignment.

**Video S6**. Improper alignment.
